# Perspectives of economic losses due to condemnation of cattle and buffalo carcasses in the northern region of Brazil

**DOI:** 10.1371/journal.pone.0285224

**Published:** 2023-05-04

**Authors:** Welligton Conceição da Silva, Raimundo Nonato Colares Camargo, Éder Bruno Rebelo da Silva, Jamile Andréa Rodrigues da Silva, Márcio Luiz Repolho Picanço, Maria Roseane Pereira dos Santos, Cláudio Vieira de Araújo, Antônio Vinicius Correa Barbosa, Marina de Nadai Bonin, Albiane Sousa de Oliveira, Simone Vieira Castro, José de Brito Lourenço

**Affiliations:** 1 Graduate program in Animal Science (PPGCAN) of the Federal University of Pará (UFPA), Castanhal, Pará, Brazil; 2 Federal Rural University of the Amazon (UFRA), Capanema, Pará, Brazil; 3 Federal Rural University of the Amazon (UFRA), Belém, Pará, Brazil; 4 Federal Institute of Education, Science and Technology of Pará (IFPA), Santarém, Pará, Brazil; 5 Federal University of Western Pará (UFOPA), Santarém, Pará, Brazil; 6 Federal University of Mato Grosso (UFMT), Sinop, Mato Grosso, Brazil; 7 Federal University of Mato Grosso do Sul (UFMS), Campo Grande, Mato Grosso do Sul, Brazil; 8 Catholic University Center of Tocantins, Tocantins, Tocantins, Brazil; Guru Angad Dev Veterinary and Animal Sciences University, INDIA

## Abstract

The work aims to study the economical losses of the condemnation of bovine and buffalo carcasses, in order to estimate the losses in animals slaughtered in Santarém-Pará, Brazil, between 2016 and 2018, with data obtained from the Municipal Department of Agriculture and Fisheries. Sex, age, origin, total number of animals slaughtered and causes of condemnation of carcasses were considered. All analyzes were performed in RStudio version 1.1.463. In this study, 71,277 bovine carcasses and 2,016 buffalo carcasses were inspected, of which 300 bovine and 71 buffalo were condemned. The highest prevalence of causes of condemnation in cattle was recorded for brucellosis (0.0020%) and tuberculosis (0.0019%). In buffaloes, tuberculosis (0.0307%) peritonitis (0,0019%) were the main causes of condemnations. Economical losses were more evident in females, for both species. The projection of economical losses related to the condemnation of carcasses showed a sharp growth for the next three years, if the average growth remains constant. The biggest projected loss was for bovine females, with an accumulated projection of $ 5,451.44. The smallest estimated loss was for buffalo males, projected at more than thirty-two thousand reais. The most important causes of condemnation report the diseases brucellosis and tuberculosis, as the ones with the greatest impact. In the buffalo species this was even more accentuated, even though the number of buffaloes slaughtered is more than 35 times smaller than the number of cattle.

## Introduction

Red meat is included in the diet of Brazilians, being a food highly sought after by the population [1–3], thus, it is necessary to inspect the meat efficiently in refrigerated slaughterhouses. Some animals can be carriers of zoonotic diseases, which can cause public health problems and generate economical losses in slaughterhouses, due to the condemnation of carcasses [4–7].

Among the main diseases that affect bovine and buffalo herds, tuberculosis [8] and brucellosis, which cause considerable economic losses [9]. The northern region of Brazil, especially the state of Pará, has the largest buffalo herd and the fifth largest cattle herd [10].

Regarding the demand, population projections point out rapid and continuous growth in the upcoming decades, which should increase food demand in general [11]. According to a report by the United Nations Population Division, it is estimated that the world’s population will reach about 8.5 billion by 2030, and for an even greater increase of about ten billion by 2050 [12].

In this context, it is essential that, in agricultural production, there is an amount of quality, healthy food, given that they need the sustainable use of scarce agricultural resources [13]. Livestock activity plays an important role in food safety, which is considered a valuable resource and a source of wealth [14].

Meat inspection in slaughterhouses, plays an important role in preventing the transmission of parasitic diseases as well as of those transmitted from animals to humans. In addition, slaughterhouse data regarding the condemnation of organs and carcasses can be analyzed to verify the rate of food waste (meat) and financial losses, due to diseases and failures in technological processing during slaughtering [15]. Based on this information, the objective of this work was to study the economic losses arising from the condemnation of bovine and buffalo carcasses, in order to estimate their future damage, in animals slaughtered in Santarém, Pará, Brazil.

## Materials and methods

### Study area

The work was carried out through a retrospective analysis over three years, from January 2016 to December 2018. Data regarding slaughtering of cattle and buffalo were collected from the Municipal Agriculture and Fisheries Department (Secretaria Municipal de Agricultura e Pesca-SEMAP), in the municipal inspection sector, in the municipality of Santarém, Pará, Brazil.

### Collection and characterization of data

Information from three slaughterhouses (A, B and C), under the control of the Municipal Inspection Service (SIM), that is, local slaughterhouses in the municipality, which follow the same guidelines and standards, aimed at inspecting the meat, with the help of qualified and experienced veterinarians, who inspect carcasses, organs and subsequently record the causes of condemnation in standardized files. Information on the condemnation was obtained through monthly reports provided in Microsoft Excel^®^ spreadsheets. Specifically, gender, age, origin, total number of slaughtered animals and causes of condemnation of carcasses due to tuberculosis, brucellosis, hematoma, cachexia, generalized lesions, jaundice, dipoxanthosis, peritonitis and contamination were considered. The sex, age and municipality of origin were identified using the Animal Transit Guide (GTA). The GTA considered an official document and mandatory issue for the intradistrict transit and interest of animals for any purpose within the Brazilian territory.

Regarding the breed of slaughtered cattle, there is no data on the proportion of dairy cattle and beef cattle, considering that in the Amazon region, breeders tend to develop the practice of mixed livestock, that is, both beef and milk, as this tends to enhance the economic gains of small producers in the interior of the Amazon. In general, the herd comes from beef herds, with a predominance of Zebu cattle, crossbreeds of the Nelore breed, with little participation of bulls and dairy cattle.

### Municipality of origin

**Fig 1:**
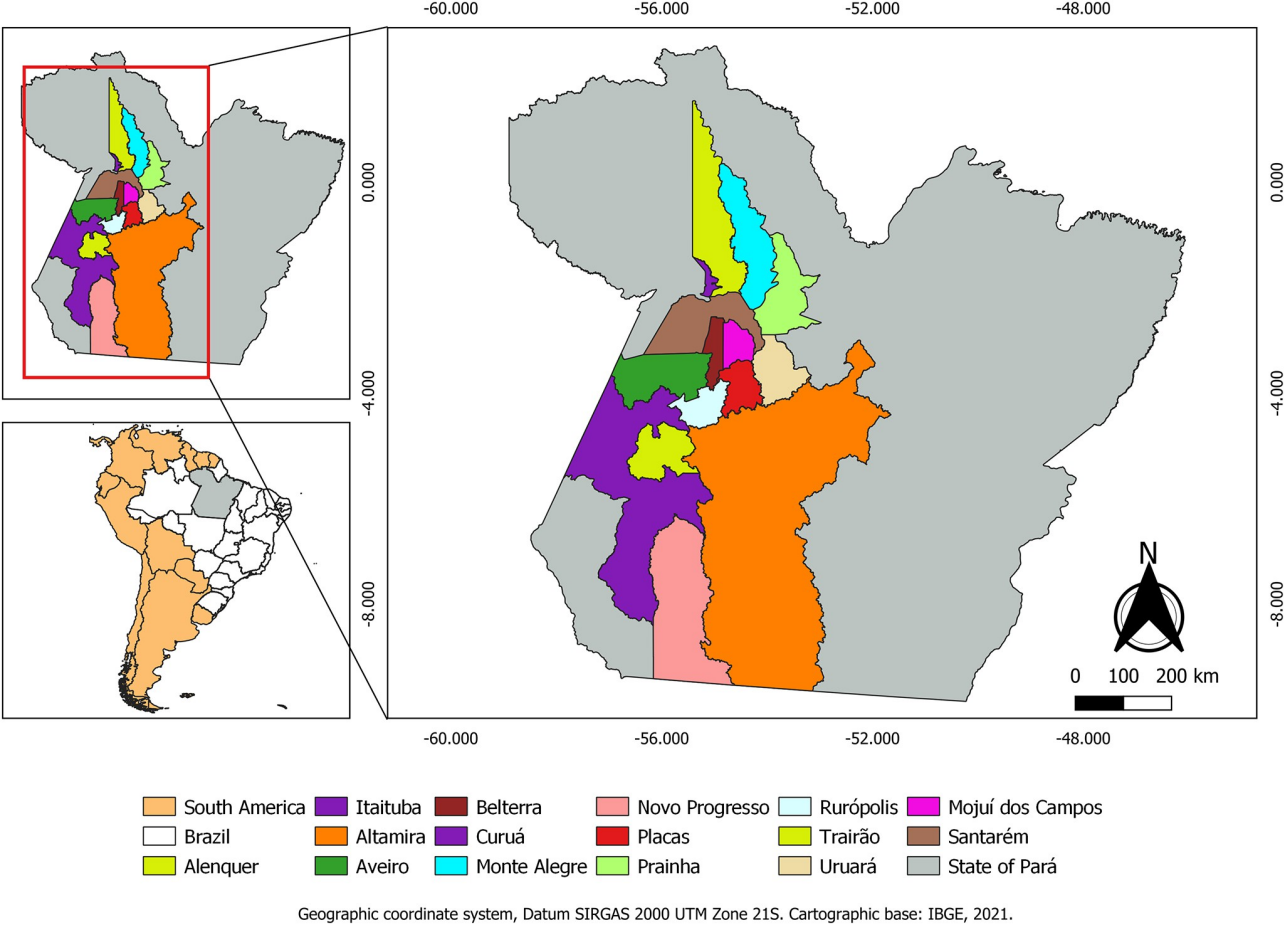
Municipalities of origin of cattle and buffalo slaughtered in Santarém, Pará, Brazil.

The animals came from 15 municipalities in Pará, Eastern Amazon, which were: Aveiro, Itaituba, Novo Progresso, Rurópolis, Trairão, Altamira and Uruará, from the Southwest Mesoregion of Pará: Alenquer, Belterra, Curuá, Mojuí dos Campos, Monte Alegre, Placas, Prainha and Santarém, in the Lower Amazon Mesoregion (Fig 1). All animals from these municipalities were slaughtered in the three slaughterhouses located in Santarém, Pará.

### *Post mortem* inspection of carcasses

In this study, 73,293 cattle and buffalo carcasses were inspected between January 2016 and December 2018. The average slaughter during the study period was 24,431 cattle/buffaloes, which is an average derived from three years of evaluation (2016 to 2018). The condemnations were carried out by the Municipal Inspection Service (Serviço de Inspeção Municipal-SIM), based on the inspection of macroscopic lesions suggestive of zoonotic diseases, present in the carcasses or in different organs evaluated (lung, lymph nodes, liver and spleen), according to Art. 171 of the RIISPOA [16]. Animals diagnosed with brucellosis and/or tuberculosis were not linked to the sanitation program for infected farms. Carcasses that had brucellosis and tuberculosis as the cause of condemnation were counted together with the other causes of condemnation.

### Cases prevalence

To calculate the prevalence, the total number of convictions for each cause of conviction in the 15 municipalities was considered, compared to the total number of animals slaughtered, using the formulas shown in Table 1. To calculate the prevalence of condemnations, first the effect of different sample sizes was eliminated, that is, the number of condemned carcasses was divided by the total number of carcasses inspected, for each month and, from there, the condemnation rate was calculated. for every thousand observations.

**Table 1:** Formulas to calculate the prevalence of causes of condemnation in the *post mortem* of carcasses.

Formula	Description of abbreviations	Illness
P_t_ = NIC_t_/ TIS	P_t_ = Prevalence of Tuberculosis NIC_t_ = Number of individuals condemned by tuberculosis TIS = Total of individuals slaughtered	Tuberculosis
P_b_ = NIC_b_/ TIS	P_b_ = Prevalence of brucellosis NIC_b_ = Number of individuals condemned by brucellosis TIE = Total of individuals slaughtered	Brucellosis
P_h_ = NIC_h_/ TIS	P_h_ = Prevalence of hematoma NIC_h_ = Number of individuals condemned by hematoma TIE = Total of individuals slaughtered	Hematoma
P_c_ = NIC_c_/ TIS	P_c_ = Prevalence of cachexia NIC_c_ = Number of individuals condemned by cachexia TIE = Total of individuals slaughtered	Cachexia
P_cg_ = NIC_cg_/ TIS	P_cg_ = Prevalence of generalized injury NIC_cg_ = Number of individuals condemned by generalized injury TIE = Total of individuals slaughtered	Generalized Injury
P_i_ = NIC_i_/ TIS	P_i_ = Prevalence of jaudice NIC_i_ = Number of individuals condemned by jaudice TIE = Total of individuals slaughtered	Jaundice
P_a_ = NIC_a_/ TIS	P_a_ = Prevalence of Adipoxantosis NIC_a_ = Number of individuals condemned by Adipoxantosis TIE = Total of individuals slaughtered	Adipoxantosis
P_p_ = NIC_p_/ TIS	P_p_ = Prevalence of Peritonitis NIC_p_ = Number of individuals condemned by peritonitis TIE = Total of individuals slaughtered	Peritonitis
P_c_ = NIC_c_/ TIS	P_c_ = Prevalence of contamination NIC_c_ = Number of individuals condemned by contamination TIE = Total of individuals slaughtered	Contamination

### Economical losses

Economical losses were estimated using information provided by beef and buffalo meat traders, in relation to the average cost of their carcasses, both for beef and buffalo meat (kilograms—kg), obtained in November 2021. Thus, the following equation was used:

CEL=ACPXTCC (1)

Where: CEL = carcass economical loss (currency/kg); ACP = average carcass price (price per kg); TCC = total condemned carcasses.

Carcass weight in kg (mean, standard deviation) was considered equivalent to male bovines of 285.4±14.25 and females 225.2±20.54, and male buffaloes 189.2±35.00 and females 217.2± 13.62. In the cost calculations, the exchange rate equivalent to USD $1 = R$5.87 reais was used.

### Projection of economical losses

The methodology used to carry out the projections of financial losses related to the bovine and buffalo carcass was the simple average, described as one of the most used in scientific studies [17]. Initially, the amount in units of carcasses lost due to technopathy (slaughter-related problems) or even disease (animals slaughtered with some type of animal disease) was raised.

Annual average of carcasses discarded per year = Number of condemned lost carcasses (technopathy/diseases) for the calculated year / number of carcasses inspected/evaluated in the calculated year (Eq 1):

$${\bar{X}}_{DC^{year}}=\;\frac{carcasses\;discarded\;\left(year\right)}{evaluated\;carcasses\;\left(year\right)}$$

After this survey of the amount of carcasses that were being discarded, the annual average of condemnation growth was calculated, according to Eq 2:

$$\text{Financial}\;\text{losses}=\left[{\left(\frac{{\bar{X}}_{DC^{firt\;year}}}{{\bar{X}}_{DC^{last\;year}}}\right)}^{\frac{1}{n}}-1\right]\times 100\%$$ (2)

Where: n equals the number of years studied in the historical series, including the initial and final year values.

With these data, it is possible to calculate the average expected losses for the years 2022, 2023 and 2024. Data for the years 2019 and 2021 were selectively excluded from the survey, as they demonstrated a reality equated in a pandemic context (Covid-19), when the consumption of animal protein had an atypical change.

### Statistical analysis

In the statistical analysis of the data, the effects of different sizes of the samples were removed, that is, the quantity of condemned carcasses was divided by the total inspection for each month, and later the condemnation rate was calculated for each thousand observations, considering then the data, for non-parametric statistical analysis, as whole numbers.

In the comparison between bovine and buffalo species, for each disease, the Wilcoxon-Mann-Whitney test was performed. Otherwise, in comparing the diseases for each species, the Kruskal-Wallis test was performed followed by the post-hoc test to compare each of the diseases, according to each species.

When comparing the months, for each species, the chi-square test was performed for the contingency table and, subsequently, a comparison test of pairs of variables. All analyzes were performed using R software version 3.4.1 (R Core Team 2016) [18].

## Results

In the three slaughterhouses, 71,277 bovine carcasses were inspected, specifically 24,751 animals in 2016, 23,127 in 2017, and 23,399 in 2018 and 2,016 buffaloes between January 2016 and December 2018, in which 903 carcasses were inspected in 2016, 549 in 2017 and 564 in 2018. Of these, 300 cattle were condemned, of which 66, 103 and 131 corresponded to the years 2016, 2017 and 2018, respectively. In the buffalo species, 71 animals were condemned, 31 in 2016, 23 in 2017 and 17 in 2018.

Regarding sex, 10, 14 and 30 male cattle were condemned in 2016, 2017 and 2018, respectively. Regarding females, 56 (2016), 89 (2017) and 101 (2018) were condemned. In male buffaloes, eight (2016), four (2017) and six (2018) convictions were recorded and 23, 27 and 11 of females in 2016, 2017 and 2018, respectively.

The highest prevalence of cause of condemnation in cattle was brucellosis (0.0020%) and tuberculosis (0.0019%). On the other hand, in buffaloes, tuberculosis (0.0307%) and peritonitis (0,0019%) were the main causes of condemnations (Table 2).

**Table 2:** Prevalence of causes of condemnation of cattle and buffalo identified in the *post mortem*.

Cause of condemnation	Bovine		Buffalo	
	Number	Prevalence (%)	Number	Prevalence (%)
Tuberculosis	137	0.0019	62	0.0307
Brucellosis	143	0.0020	2	0.0009
Hematoma	2	0.00002	-	-
Cachexia	1	0.000014	1	0.0004
Generalized Injury	12	0.00016	-	-
Jaundice	5	0.000070	-	-
Adipoxantosis	1	0.000014	-	-
Peritonitis	1	0.000014	4	0.0019
Contamination	2	0.00002	-	-

Note: p-value less than 0.05 indicates statistical difference.

Tuberculosis and brucellosis were the diseases that most caused condemnation rates in cattle, respectively, with a rate of 234 per 1,000 inspections, and therefore did not differ from each other (p>0.05) (Table 3). In the buffalo species, the rates of condemnation for tuberculosis were much higher than the other causes (p>0.05). For both cattle and buffaloes, there were no differences between the other causes of condemnation, however, the highest rates were observed in generalized injury, with a rate of 20 cases per 1,000 inspections and peritonitis with 13 cases per 1,000 inspections, respectively.

**Table 3:** Comparison between the causes of condemnation for cattle and buffaloes.

Case of condemnation	Bovine	Buffalo
Tuberculosis	234a	398a
Brucellosis	245a	12b
Hematoma	4b	0b
Cachexia	2b	2b
Generalized Injury	20b	0b
Jaudice	9b	0b
Adipoxantosis	2b	0b
Peritonitis	2b	13b
Contamination	4b	0b

Note: Different letters (a, b) in the column indicate statistical differences (p<0,05).

In the bovine species, the months of April (39 cases), May (40 cases) and December (32 cases) had the highest rate of condemnations, not differing from each other (p>0.05), but from the other months (p<0.05). The other months did not show differences among themselves in the species (p>0.05). In buffaloes, the highest number of cases occurred in June and December, both with 52 cases, differing from the other months of the year (p<0.05) (Table 4).

**Table 4:** Comparison between the causes of condemnation for cattle and buffaloes in terms of months.

Cases of condemnation	Bovine	Buffalo
January	10ac	30 ab
February	11acd	27 ab
March	13 acd	27 ab
April	39 b	17 ab
May	40 b	43 be
June	20 ad	52 c
July	10 ac	36 bce
August	11 acd	0 d
September	18 acd	35 bce
October	9 c	37 bce
November	21 d	41 bce
December	32 b	52 e

Note: Different letters (a, b, c and d) in the column indicate statistical differences (p<0,05).

There were differences (p<0.05) between the number of cattle and buffaloes condemned for tuberculosis, brucellosis, generalized injury and jaundice, all these causes being more evident in the bovine species. The other causes did not differ between species (p>0.05) (Table 5).

**Table 5:** Comparison between the different causes of carcass condemnation between bovine and buffalo species, regardless of year and months.

Causes of condemnation	p-value
Tuberculosis	0.000592
Brucellosis	0.0003034
Hematoma	0.3593
Cachexia	1
Generalizada Injury	0.002856
Jaundice	0.03669
Adipoxantosis	0.3593
Peritonitis	0.2478
Contamination	0.3593

Note: p-value less than 0.05 indicates statistical difference.

Regarding the projections for economic losses, the first calculation was for the male bovine group, which had an average growth increase in the three years of the research of 97.14%, then it was calculated from bovine females with an average growth of 65.67%, the later was calculated from male buffaloes that had an average negative waste growth of -25%, and finally the average growth of female buffaloes which also had a negative average of 12.24% was calculated.

Economical losses for male cattle increased by 32.66%, from 2016 to 2017, equivalent to $3,889.60 dollars, and 53.34%, from 2017 to 2018, with a total of $13,613.62 dollars. When evaluating from 2016 to 2018, there was an increase of 68.58%. Females showed a subsequent increase of 37.08%, from 2016 to 2017, 11.89%, from 2017 to 2018, and from 2016 to 2018, of 44.56%, that is, $20,889.43 dollars; $7,596.18 dollars and $28,144.97 dollars.

In male buffaloes, there was a reduction of 50%, that is, of $2,127.29 dollars, between 2016 and 2017, and an increase of 33.34% ($1,063.64 dollars), from 2017 to 2018, and reduction of 25%, from 2016 to 2018. The female buffaloes showed a drop in economic losses of 14.82%, that is, $2,294.10 dollars between 2016 and 2017, there was also a reduction, between 2017 and 2018, with percentage of 59.26%, equivalent to $9,176.42 dollars. These economic losses, both in cattle and buffaloes, were more evident in females. The female bovines caused 70.81% more, when compared to males of the same species, equivalent to $110,261.97 dollars more. Female buffaloes were responsible for 72.64% of the financial losses, equivalent to $25,412.29 dollars more (Table 6).

**Table 6:** Economical losses of carcass condemnation in bovine and buffalo species, by sex, in 2016, 2017 and 2018.

American dólar				
Year of condemnation	Bovine		Buffalo	
	Male	Female	Male	Female
2016	$8,022.32	$35,448.86	$4,254.58	$13,191.11
2017	$11,911.93	$56,338.36	$2,127.29	$15,485.21
2018	$25,525.55	$63,934.55	$3,190.94	$6,308.79
Total	$45,459.80	$155,721.77	$9,572.81	$34,985.11

Note: USD = R 5,87 reais.

The projection of economic losses related to the condemnation of carcasses showed a sharp increase in financial losses for the next three years, that is, 2022, 2023 and 2024, if the average loss growth remains stable. The largest projected losses are for the female bovine species, with a cumulative projection of losses of more than three million reais (Table 7). The smallest loss evidenced was for the male buffalo species, with an accumulated projected loss of more than thirty-two thousand reais.

**Table 7:** Condemned animal data by year and economical loss projections for the years 2022 to 2024.

Data per year	Condemned male bovine (UN)	Condemned female bovine (UN)	Condemned male buffalo (UN)	Condemned female buffalo (UN)
2016	10	56	8	23
2017	14	89	4	27
2018	30	101	6	11
Annual growth rate				
2016 TO 2017	40,00%	58,93%	-50,00%	17,39%
2017 TO 2018	114,29%	13,48%	50,00%	-59,26%
Average annual growth rate				
2016 TO 2018	97,14%	65,67%	-25,00%	-12,24%
Projection of losses in units for the next three years				
2022	59	167	5	10
2023	117	277	3	8
2024	230	459	3	7
Average price per kg paid for products				
2016	$2,98	$2,81	$2,81	$2,64
2017	$2,98	$2,81	$2,81	$2,64
2018	$2,98	$2,81	$2,81	$2,64
Average weight of animals in kg				
2016	285,4	225,2	189,2	217,2
2017	285,4	225,2	189,2	217,2
2018	285,4	225,2	189,2	217,2
Projection of financial losses for the next three years				
2022	$50,321.07	$105,920.36	$2,393.20	$5,536,59
2023	$99,202.97	$175,478.27	$1,794.90	$4,858.91
2024	$195,568.73	$290,714.85	$1,346.17	$4,264.18
**Total**	$345,092.78	$572,113.49	$5,534.28	$14,659.69

UN = unit, $ = 5,87.

## Discussion

A higher rate of convictions was found in cattle, which may be associated with inadequate management carried out on farms in the region studied.

Tuberculosis, the main cause of condemnation and, consequently, of financial losses, as also described by Noronha et al. [19], with a prevalence of less than 0.04% and 0.002%, in buffaloes and cattle, respectively, with a prevalence of buffaloes, more than sixteen times higher than that of cattle, are far below that reported by Kantor and Ritacco [20], of 0.37% prevalence of tuberculosis in the animals studied in the Midwest region of Argentina.

The results found in the present study corroborate the work developed in the municipality of Santarém, Pará, by Pereira et al. [21], who identified a higher occurrence of tuberculosis in bovine females (87.6%) and buffaloes (71.4%). Females are more likely to contract the disease. This can be explained by the fact that females are more likely to contract the disease, because they normally spend more time in the herd and, consequently, suffer greater exposure to the agent [22]. On the other hand, the excessive slaughter of matrices and the subsequent increase of young animals in the herd may reflect in the decrease in the number of calves [23]. Thus, the renewal of matrices within the herd is essential for the genetic improvement of the herd, provided that these animals are replaced by others with high productive potential [24].

The lower number of infections in males may be related to the fact that these animals are isolated from other animals for most of the year, making it possible to identify a higher proportion of infected bulls when compared to young animals [25]. In a study carried out by Oliveira et al. [26] a prevalence of 0.003% was identified in the carcasses of cattle affected by tuberculosis in the state of Maranhão. Lopez et al. [27] identified a prevalence of 0.00087% in the state of Rondônia due to tuberculosis.

When related to age, it was identified that more than half (55.1%) of the animals were older than seven years, which can be explained by the chronic nature of the disease, especially in older animals. Pereira et al. [28] identified that the largest number of positive bovines and buffaloes were older than seven years, followed by younger animals aged 0 to three years, as these young animals may have acquired the disease through the ingestion of milk and/or contaminated colostrum [29].

Brucellosis was one of the main causes of condemnation in cattle, also evidenced to a lesser extent, cases of condemnation in buffaloes [30]. This disease has been reported in several countries [31–35], with the main means of contamination in cattle, the digestive, as well as it is contracted during the breeding period, both by natural mating, but especially by artificial insemination [36]. It is noteworthy that the proliferation of the epidemiological agent in ruminants occurs mainly through infected females, with or without the presence of a previous history of abortion [37, 38]. In this way, the slaughter of females on a large scale is a worrying factor, since it may provide the livestock industry with a reduction in the production of calves.

A prevalence of 0.002% was found, a lower rate than that reported by Sousa et al. [39], who found a 1,19% prevalence of brucellosis in cattle in the state of Maranhão, Brazil. In a study carried out by Freitas and Oliveira [38], the prevalence of bovine brucellosis was 0.099%, much higher when compared to the results found in the present study, while Roma et al. [34] identified a lower prevalence of 0.001% [42/23.963].

Carcass condemnation by hematoma was evidenced in cattle and absent in buffaloes, possibly as a result of bad habits in the transport of cattle, such as acceleration, sudden braking during access to poorly planned highways, which affect a series of injuries to the animal, by falling of cattle inside the truck and lack of proper direction by the driver [40]. In addition, other animals trample them, causing bruises on a large part of the body. The removal of injured meat parts was on average 0.3 kg of discarded meat per carcass. These results are similar to those described contrary to the average described by Melo et al. [41], with a loss of 2.33 kg, with a total loss of 1,143.8 kg, in 490 animals. When comparing batches of cattle transported on paved and unpaved roads, the occurrence of a higher rate of injuries in animals transported on unpaved roads over long distances is noticeable [42].

Cachexia is a disease that is associated with the popular tuberculosis, becoming a determining factor in the total condemnation of the carcass [16]. Similar results were obtained by Yibar et al. [43], who verified the prevalence of tuberculosis and cachexia as one of the main causes of condemnation.

The clinical signs of cachexia manifested in bovines is one of the reasons for condemnations, as established by RIISPOA in Art. 113, paragraph 2. Cachexia is characterized as complex, and not easily defined, being considered a metabolic syndrome, consisting of progressive weight loss [44, 45].

Injury rates are associated with inadequate handling of cattle, whether in loading, transporting or unloading. The greater number of wounds in the rear regions of cattle, which affect the quality of the meat, is possibly due to the use of inappropriate objects, such as shock sticks and pointed pieces, while driving the animals [45–47]. During loading and unloading, the lack of training of employees can cause disordered movement in animals, as well as stress [48–50], which which favors the appearance of injuries [51–53].

In a study carried out in California, the presence of peritonitis was identified as one of the causes of the increase in condemnation of contaminated carcasses. For White et al. [54] and Dupuy et al. [55], the emergence of these condemnations may be the result of certain aspects in all parts of the production chain, such as environmental conditions, management, stress and accommodation of pathogens in bovine animals. Corroborating the reports by Bassuino et al. [56], who describe peritonitis as a very evident disease in production animals, although little known by most rural owners.

It is noteworthy that in the region where this study was developed, it is common for slaughterhouses to receive animals from neighboring municipalities. Therefore, it is important to clarify to producers the main causes of condemnation of carcasses and to draw their attention to the damage resulting from this practice. All of this will encourage the tracking of sick animals at source, in addition to mitigating management practices that may promote condemnation of carcasses.

On the other hand, contamination of carcasses is identified as one of the forms of condemnation. The main causes of condemnation of carcasses are related to diseases, in about 57%, especially tuberculosis [10] and brucellosis [35, 57].

Another possible means of contamination is the incorrect washing of the animals, as it dumps external impurities adhered to the skin, in addition to cleaning the anus and extremities region, to avoid contamination of the carcasses in later stages [58]. According to Picchi [59], in addition to the fur, the flow of blood clots in the carcass cavities contributes to the proliferation of microorganisms, which demonstrates the importance of washing the carcass, as one of the forms of elimination for possible contamination foci.

In relation to economical losses, it was evident that they presented expressive values in this study. The paucity of studies in this area is highlighted and this is even worse when it comes to research carried out in the Southwest Paraense and Lower Amazon Mesoregions.

Therefore, the work developed by Dourado et al. [10] deserves attention, where a loss caused by tuberculosis in cattle was observed, in the order of $ 50,638.96, of which approximately 58% resulted from the condemnation of females.

From another perspective, still in relation to the damage caused by post-mortem condemnation in cattle developed in the region of this research, there is the work of Pereira et al. [15], which alleges economic loss of $ 41,116.30 subsequent to the condemnation of tongue, liver and heart, for several causes. On the other hand, unfortunately, there were no studies with buffaloes, making it impossible to compare data.

It is noteworthy that there are no studies estimating financial projections for losses due to carcass condemnation in cattle and buffaloes. Thus, this investigation proved to be a pioneer for this methodology and for this reason there are no data on this aspect that can be compared with the results obtained in this investigation.

## Conclusions

The estimate of economical losses resulting from the condemnation of estimated carcasses points to a severe increase for the next three years, especially for those from buffalo carcasses. Among the protagonists responsible for the worsening of the damage, the diseases brucellosis and tuberculosis stand out. This forecast may even discourage the slaughter of buffaloes, perhaps even the beef buffalo culture. In this way, more important than quantifying the economic damage, it is to work towards mitigating the expected losses in the short term, because from the knowledge of factors causing damage, the more assertive the strategy can be to minimize their impacts or even avoid them.
